# Iatrogenic Cushing's Syndrome with Subsequent Adrenal Insufficiency in a Patient with Psoriasis Vulgaris Using Topical Steroids

**DOI:** 10.1155/2017/8320254

**Published:** 2017-11-13

**Authors:** Suzan Demir Pektas, Gursoy Dogan, Nese Cinar

**Affiliations:** ^1^Department of Dermatology, Mugla Sitki Kocman University, Faculty of Medicine, 48000 Mugla, Turkey; ^2^Department of Endocrinology and Metabolic Diseases, Mugla Sitki Kocman University, Faculty of Medicine, 48000 Mugla, Turkey

## Abstract

Iatrogenic Cushing's syndrome (ICS) is usually related to prolonged and/or high-dose oral or parenteral steroid use. Psoriasis vulgaris (PV) is chronic inflammatory disease and characterized by periods of attack and remission. Topical steroid (TS) is the first choice of treatment for localized and mild PV. The development of systemic side effects of the steroids is usually not observed after TS application. But the risk of developing ICS still exists. In the literature, there are a few adult cases who developed ICS and subsequent adrenal insufficiency associated with TS. In this article, a male patient with PV developing ICS and secondary adrenal insufficiency after treatment of TS for 12 years is presented.

## 1. Introduction

Iatrogenic Cushing's syndrome (ICS) is usually related to prolonged and/or high-dose oral or parenteral steroid use [1]. The development of systemic side effects after topical steroid therapy is rare, but the risk of developing ICS still exists. In the literature, patients with ICS associated with topical steroids (TS) are more common in children [2, 3]. Since children have thinner dermis layer of epidermis than adults with a higher surface/volume ratio steroids are more absorbed through the skin of children leading to systemic side effects [2, 3]. In the literature, there are a few adult cases who developed ICS and secondary adrenal insufficiency associated with TS [1, 4, 5]. In this case report, a male patient with psoriasis vulgaris (PV) developing ICS after treatment of TS for 12 years is presented.

## 2. Case Presentation

**Figure 1:**
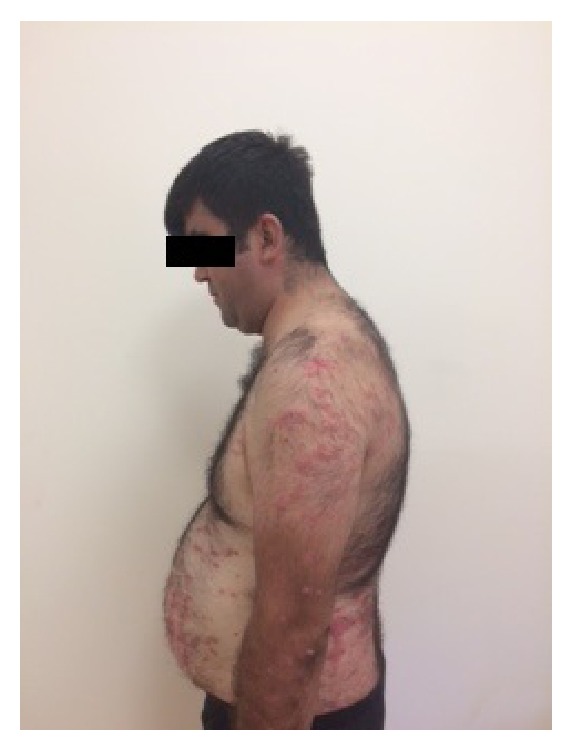
He had a moon face appearance with truncal obesity, buffalo hump, hypertrichosis in trunk, and purple striae in the axilla, periumbilical, and inguinal region.

**Figure 2:**
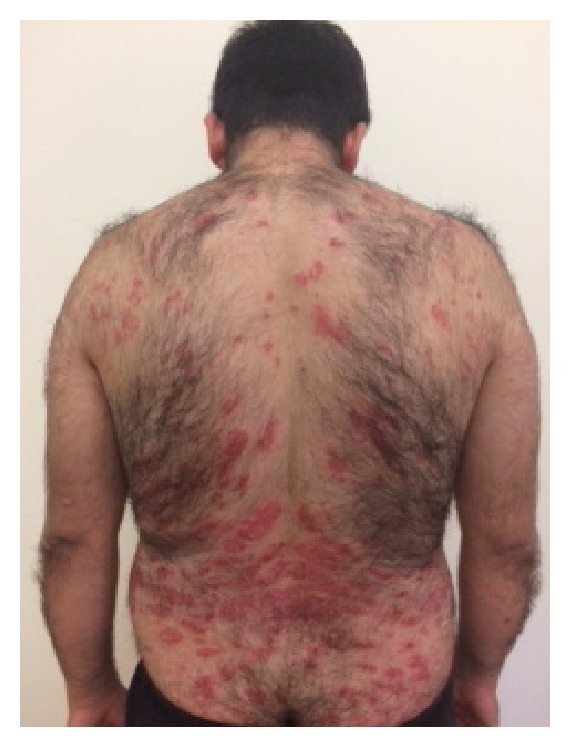
He had numerous erythematous, scaly psoriatic plaques located on the shoulders, all extremities, dorsum of hands, and feet and at the intertriginous areas.

A 32-year-old man was referred to the outpatient clinic of the Department of Endocrinology and Metabolism in Mugla Sitki Kocman University Training and Research Hospital with complains of excessive weight gain and muscle weakness. The patient gained 8 kgs in 12 months. He had a history of PV for 12 years. He used clobetasol propionate (CP) 0.05% ointment continuously at 150 mg/week for the past 12 years and stopped the steroid ointment treatment a month ago by himself. On physical examination, he had a moon face appearance with truncal obesity, buffalo hump, hirsutism, and purple striae in the axilla, periumbilical, and inguinal region (Figure 1). Numerous erythematous, scaly psoriatic plaques were located on the shoulders, all extremities, dorsum of hands, and feet and at the intertriginous areas (Figure 2).

Vital signs revealed an arterial blood pressure of 130/80 mmHg, pulse rate 75/min, height 172 cm, weight 85 kg, and body mass index 28.7 kg/cm^2^. On laboratory studies, liver and kidney function tests and fasting blood glucose were within normal limits. The following were found: morning adrenocorticotropic hormone (ACTH): 17 : 53 pg/ml (*N*: 7.2–63.3); morning basal cortisol: 3.83 mg/dL (*N* = 6.24–18.0). Other anterior hypophyseal hormones were as follows: thyroid stimulating hormone (TSH): 2.77 *μ*/ml (*N*: 0.27–4.2); fT4: 17.6 pmol/L (*N*: 12–22); fT3: 7.26 pmol/L (*N*: 3.1–6.8); prolactin: 7.34 ng/ml (*N*: 4.04–15.2); follicle stimulated hormone (FSH): 3.81 mIU/mL; luteinizing hormone (LH): 2.7 mIU/mL; total testosterone: 2.77 ng/mL (*N*: 2.9–8.36). 1 *μ*g ACTH stimulation test was performed to evaluate hypophyseal-adrenal axis and there was insufficient response to the test (peak cortisol level 7.7 mg/dL). Clinical symptoms and laboratory findings of the patient suggested the diagnosis of ICS due to the prolonged use of a high-potent TS with subsequent ACTH deficiency. The patient had osteopenia on dual energy X-ray absorptiometry (DEXA) scan of the spine. The topical steroid was ceased, and methotrexate (10 mg/week) for psoriatic plaques and hydrocortisone acetate (20 mg/m^2^/d) were prescribed in order to prevent adrenal insufficiency. Calcium and vitamin D supplementation were given for osteopenia. Calcipotriene ointment and moisturizing lotion treatment were also started.

## 3. Discussion

Local adverse effects of topical steroids are acne, purpura, atrophy, striae, and telangiectasia. ICS and hypothalamic-pituitary-adrenal (HPA) axis suppression are rare but unignorable complications of topical steroids [1, 4–6]. ICS patients may present with purple striae, central obesity, hypertension, buffalo hump, muscle weakness, moon face, hirsutism, and easy bruising of skin like in our patient [1, 4–6].

PV is chronic inflammatory disease and characterized by periods of attack and remission [4–6]. TS is the first choice of treatment for localized and mild PV [4, 5]. In the literature, patients with ICS associated with TS are more frequent in the pediatric age group due to thinner dermis layer of skin and higher surface/volume ratio leading to more absorption rate through the skin [2, 3]. ICS rarely occurs through the topical administration in adults [1, 4, 5]. In the literature, the risk of developing steroid-induced ICS is reported to be associated with the age of the patient, size and localization of lesions, the epithelium integrity, individual metabolic variation, duration of use, powder of drug, duration of therapy, and the sensitivity of steroid receptors [1]. CP binds with high affinity to steroid receptors, with a long half-life making it an effective agent. Sahip et al. [4] presented an adult patient who developed ICS after the treatment of CP 0.005% for 10 years in psoriasis. It was reported in a review that 43 cases including children and adults developed ICS after very potent topical steroid use (majority CP) [7]. The majority of the patients in the children group (*n*: 22) had diaper dermatitis (86%). The rest of the children (27%) had disorders such as skin dryness, psoriasis, and burn. Mostly used steroid agents in child group were CP and betamethasone. The mean duration of use was 2.75 months (1–17 months). Almost all cases showed the specific Cushing's features. Psoriasis was the most commonly observed disorder in the adult group (71%) as it is in our case. The other diseases were xerosis, lichen planus, intertrigo, dermatitis, and skin lightening (19%). Similar to the group of children, CP 0.05% was the mostly used agent in adult group. The duration of steroid use in adults was about 18 months (0.3–84 months). In this review, it was reported that the suppression of the HPA and secondary adrenal insufficiency in children group was more prominent than in adult group because of multiple risk factors written above [7]. The use of highly effective topical corticosteroids, the amount, frequency, and duration of the application, the structure and the area of the affected skin, and the application of steroids with absorbent clothing were among the main risk factors for the development of ICS after topical steroid use. Additionally, in some countries, individuals can easily buy steroid-containing topical products without a doctor's prescription. Our patient has expressed that steroid ointments were easily purchased in our country.

Moreover, most of these risk factors also play a role in the development of ICS and subsequent adrenalin sufficiency in our patient since he has been using a potent TS (CP) on a great area of the body with large lesions for a very long period of 12 years.

Allenby et al. [8] studied the effects of CP on the HPA axis in 39 patients and found that when more than 50 g per week of CP was applied to the skin, adrenal suppression was to be expected. On the other hand, Sibel et al. [6] showed that four patients (three psoriasis, one atopic dermatitis), who used CP 0.005% cream over a long-term period, developed secondary adrenal deficiency almost 4 months after withdrawal of treatment though three of them used less than 50 g topical steroid per week. Even relatively small doses of CP cream may disrupt the HPA axis, occurring more commonly than has previously been recognized. Following prolonged use of CP at medium doses, adrenal insufficiency may be more common than previously described.

There is no consensus regarding withdrawal of the steroid treatment in case of development of ICS [1, 4, 6]. The three major complications that may develop upon the cessation of the steroid treatment are exacerbation of the underlying disorder, secondary ACTH failure, and steroid withdrawal syndrome [1, 4, 6, 9]. When CP 0.05% ointment has been used over 2 g per day, it reduces morning cortisol levels after a short time [3, 7]. Tempark et al. [7] reported that the estimated period of the amelioration of the HPA axis disruption was 3.49 ± 2.92 months in children and 3.84 ± 2.51 months in adults. Sibel et al. [6] reported that four patients had evidence of secondary adrenal failure following withdrawal of CP or considerable lowering of the dose, and the HPA axis activity returned normal after 1–4 months. They suggested that when topical CP is used chronically, it should be kept in mind that secondary adrenal failure is likely to occur even though small amount of steroids (7.5 g per week) is used [10]. The dose of glucocorticoids should be increased in case of intervening infections or any operation causing major stress in the patient. This procedure should be applied while using topical steroids or in the 4 months' period after cessation of the treatment. The topical steroid was ceased, and methotrexate (10 mg/week) for psoriatic plaques and hydrocortisone acetate (20 mg/m^2^/d) per oral were initiated in order to prevent adrenal insufficiency in our case.

It is difficult to determine the safe dose of steroids. It has been recommended not to exceed four weeks of topical use of steroids, 50 grams per week in adults and 15 grams per week in children [6]. The cut-off value of the CP leading to the HPA axis suppression and Cushing's syndrome is not yet determined. Therefore, further studies are needed to find out the extent of absorption of steroids [1]. Which patients are more prone to the development of adrenal insufficiency during topical steroid application is still not clear. A few months after steroid treatment, we recommend investigation of ACTH and cortisol levels to evaluate adrenal function in all patients. Additionally, all patients should be informed about the characteristic Cushing's syndrome sign and symptoms, for earlier referral to the doctor to diagnose the systemic side effects of glucocorticoids such as hyperglycemia and hypertension.
